# End-Users’ Product Preference Across Three Multipurpose Prevention Technology Delivery Forms: Baseline Results from Young Women in Kenya and South Africa

**DOI:** 10.1007/s10461-017-1911-6

**Published:** 2017-10-20

**Authors:** Rachel Weinrib, Alexandra Minnis, Kawango Agot, Khatija Ahmed, Fred Owino, Kgahlisho Manenzhe, Helen Cheng, Ariane van der Straten

**Affiliations:** 1 Women’s Global Health Imperative, RTI International, 351 California St Suite 500, San Francisco, CA 94104 USA; 2 School of Public Health, University of California, Berkeley, CA USA; 3 Impact Research and Development Organization, Kisumu, Kenya; 4 Setshaba Research Centre, Soshanguve, South Africa; 5 Department of Medicine, Center for AIDS Prevention Studies, University of California San Francisco, San Francisco, CA USA

**Keywords:** Multipurpose prevention technologies, Acceptability, End-user research, HIV prevention, Product preference

## Abstract

A multipurpose prevention technology (MPT) that combines HIV and pregnancy prevention is a promising women’s health intervention, particularly for young women. However, little is known about the drivers of acceptability and product choice for MPTs in this population. This paper explores approval ratings and stated choice across three different MPT delivery forms among potential end-users. The Trio Study was a mixed-methods study in women ages 18–30 that examined acceptability of three MPT delivery forms: oral tablets, injections, and vaginal ring. Approval ratings and stated choice among the products was collected at baseline. Factors influencing stated product choice were explored using multivariable multinomial logistic regression. The majority (62%) of women in Trio stated they would choose injections, 27% would choose tablets and 11% would choose the ring. Significant predictors of choice included past experience with similar contraceptive delivery forms, age, and citing frequency of use as important. Ring choice was higher for older (25–30) women than for younger (18–24) women (aRR = 3.1; p < 0.05). These results highlight the importance of familiarity in MPT product choice of potential for variations in MPT preference by age.

## Background

Preventing HIV and unintended pregnancy are key health priorities for women, particularly in sub-Saharan Africa (SSA), where rates of HIV infection are at least twice as high among young (ages 18–30) women as among young men, and 59% of people living with HIV are women [1]. Likewise, in SSA, 40–60% of pregnancies are unintended [2–5].

Multipurpose prevention technologies (MPTs) are biomedical interventions that provide protection from both sexually transmitted infections, such as HIV, and unintended pregnancy. A dual-purpose product that combines HIV and pregnancy prevention could offer advantages over single-indication products. In recent population-based surveys, 4 in 10 women in Kenya and 89% of women ages 18–24 in South Africa were current users of modern contraceptive methods [6, 7]. A contraceptive product that also confers protection against HIV could lead to greater coverage of prevention methods in these countries than a single-purpose HIV prevention method. Women may face fewer barriers to using an MPT than an HIV prevention product, including community stigma around HIV, inconsistent adherence due to the need to use two prevention products, and challenges in communicating about HIV prevention with their partners [8, 9]. However, these MPT products will only be effective in reducing rates of HIV and unintended pregnancy if they are acceptable to the women who most need them.

In recent years, gaining a better understanding of end-user acceptability has emerged as a crucial component of research on new HIV prevention products for women. Acceptability among potential end-users is an important driver of product uptake, adherence (initiation and correct use), and persistence or continuation [10, 11]. Several promising HIV prevention products have failed to show effectiveness in large phase III trials conducted in Africa. These results are explained in part by low adherence to product use among trial participants, particularly among young women who comprise a key priority population for HIV and pregnancy prevention [12–14]. Adherence challenges emphasize with greater urgency the need to conduct acceptability research with young women to inform product development and roll out. Ultimately, incorporating end-user input will maximize the chances that technologies that move forward into efficacy trials, and eventually become available prevention options, are adopted and used correctly and consistently by young women.

Factors affecting prevention product choice and acceptability have been explored in many studies. In this body of research, acceptability has been conceptualized more broadly than attitudes toward the specific features of a product. Contextual factors related to the individual end-user, such as education and socioeconomic status, play an important role as predictors of acceptability, choice, and uptake [15, 16]. Multiple dimensions of acceptability for microbicides and oral pre-exposure prophylaxis (PreP) have been described. Mensch, et al. [11], proposed a conceptual model in which product choice is informed by *product acceptability factors*, based on product features, and *influencing factors* centered on the end-user and her social context. This conceptual framework underlies the research questions that informed the design of the TRIO Study, a prospective clinical acceptability study focusing on three placebo MPT delivery forms among young women aged 18–30 years: oral tablets, vaginal ring, and injections. The TRIO Study, conducted in Kenya and South Africa, targets a gap in knowledge regarding acceptability of MPTs. The research focuses on relative preference of three product delivery forms that could support a MPT indication, by testing uptake, use, choice, and preference based on women’s actual experiences with the products.

The three placebo delivery forms chosen for the TRIO Study represent promising potential MPTs as they capitalize on effective contraceptive methods already widely used by women, and reflect directions currently pursued with HIV and MPT products currently under development. All three are relatively advanced as HIV prevention methods in the product development pipeline, with the ring having completed phase III trials [17, 18], injections currently undergoing clinical trials [19], and oral PrEP tablets recently approved for use in both Kenya and South Africa [20, 21]. This paper examines product preferences before using an MPT, reflecting a real-world situation in which potential end-users would need to choose between a menu of different MPT products at a clinic or pharmacy.

## Methods

The TRIO Study was a multicomponent mixed-methods study that examined acceptability of three potential multipurpose prevention technology (MPT) delivery forms: daily oral tablets, two monthly injections, and a monthly vaginal ring. Here we report baseline results from the clinical component of TRIO a prospective randomized study in which women tried each product for one month and then chose one product to use for 2 months.

### Study Products

**Fig. 1:**
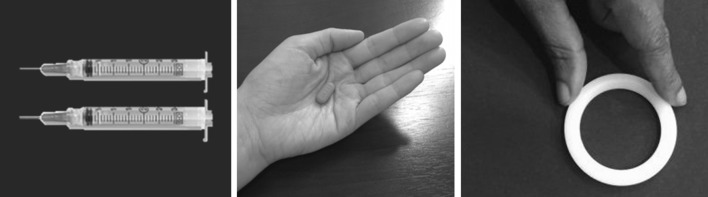
TRIO Study Products

Because the goal of the study was to investigate acceptability and use of each delivery form, uncoupled from potential side effects or effectiveness, only placebo products were used. The delivery forms are shown in Fig. 1. The MPT tablets, injections, and vaginal ring were represented by placebo Truvada (to represent a co-formulated oral tablet), two 2 mL saline injections, one in each gluteal muscle (placebo injections used in the phase III HPTN-076 trial [22]), and the silicone elastomer placebo vaginal ring developed by the International Partnership for Microbicides (used in the phase III MTN-020 ASPIRE and IPM ring studies [17, 23]), respectively. The MPT injections were presented with a monthly dosing regimen for the TRIO study with the possibility of the active product being given once every 2 months in the future. The MPT tablet, represented by placebo Truvada, was presented with a daily dosing requirement. The ring was presented with a requirement of being inserted in the vagina continuously for 1 month and then removed and replaced with a new ring.

### Eligibility and Recruitment

The clinical component of the study enrolled 277 sexually active, non-pregnant, HIV negative women ages 18–30 in Kisumu, Kenya and Soshanguve, South Africa. Study enrollment took place between December 2015 and June 2016. To be eligible for the study, women could not have participated in any prior HIV-prevention or MPT product trials or other demonstration studies.

Participants were recruited from peri-urban communities surrounding the research clinics using community mobilization and sensitization meetings, street recruitment from shopping areas, and (in South Africa only) outreach at family planning clinics and voluntary HIV counseling and testing centers. A major consideration in the study’s recruitment procedures and design was the goal of minimizing social desirability issues in reporting, such as feeling ashamed to admit nonuse or difficulty using study products, faced by other HIV prevention studies [24]. To this end, we designed multiple approaches to achieve high participant engagement in the research and encourage participation as an opportunity to contribute as a “co-designer” in the process of product development. Introductory engagement workshops, lasting around 2 h, were conducted with groups of approximately 15–30 women prior to screening visits. The content of the workshops encouraged women to view themselves as research partners and co-designers in the study, framed the study participants as a key source of feedback for product developers, and emphasized the value of honest feedback on the products, including their dislikes and negative experiences.

### Procedures

Prior to the start of the clinical acceptability study, we conducted formative interviews with 15 young women from the target population at each site, including cognitive interviewing techniques, to pretest product attribute descriptions and rating and ranking questions planned for the main study. Formative findings increased confidence that participants would comprehend the various acceptability questions, and provided insight into which product attributes were salient for these women when considering different prevention options.

To capture study-naïve product acceptability at the clinical study baseline visit, women were first shown images of the three TRIO products with minimal explanation from the interviewers. They were then asked to provide their opinions, preferences, and stated (hypothetical) product choice among the three products, using a standardized survey. Next, participants watched a brief animated educational video that explained how to use each product, including dosing frequency, and answered a subsequent set of identical questions to measure whether additional product information provided through the video changed their opinions. The video was intended to play the role of a provider who would briefly explain at the clinic how the products work. We used the format of a brief video to ensure the information would be standardized across all participants.

At the start of the acceptability questionnaire, participants were asked to imagine that they were at risk of having an unplanned pregnancy and getting HIV, and to assume that the TRIO products provided the same level of protection as condoms. Women also completed detailed demographic questionnaires at the baseline visit. Participants were then randomly assigned to one of six product-use sequences, and returned to the clinic to complete acceptability questionnaires each month. This paper presents results drawn from the baseline demographic and acceptability questionnaire data.

### Measures

**Fig. 2:**
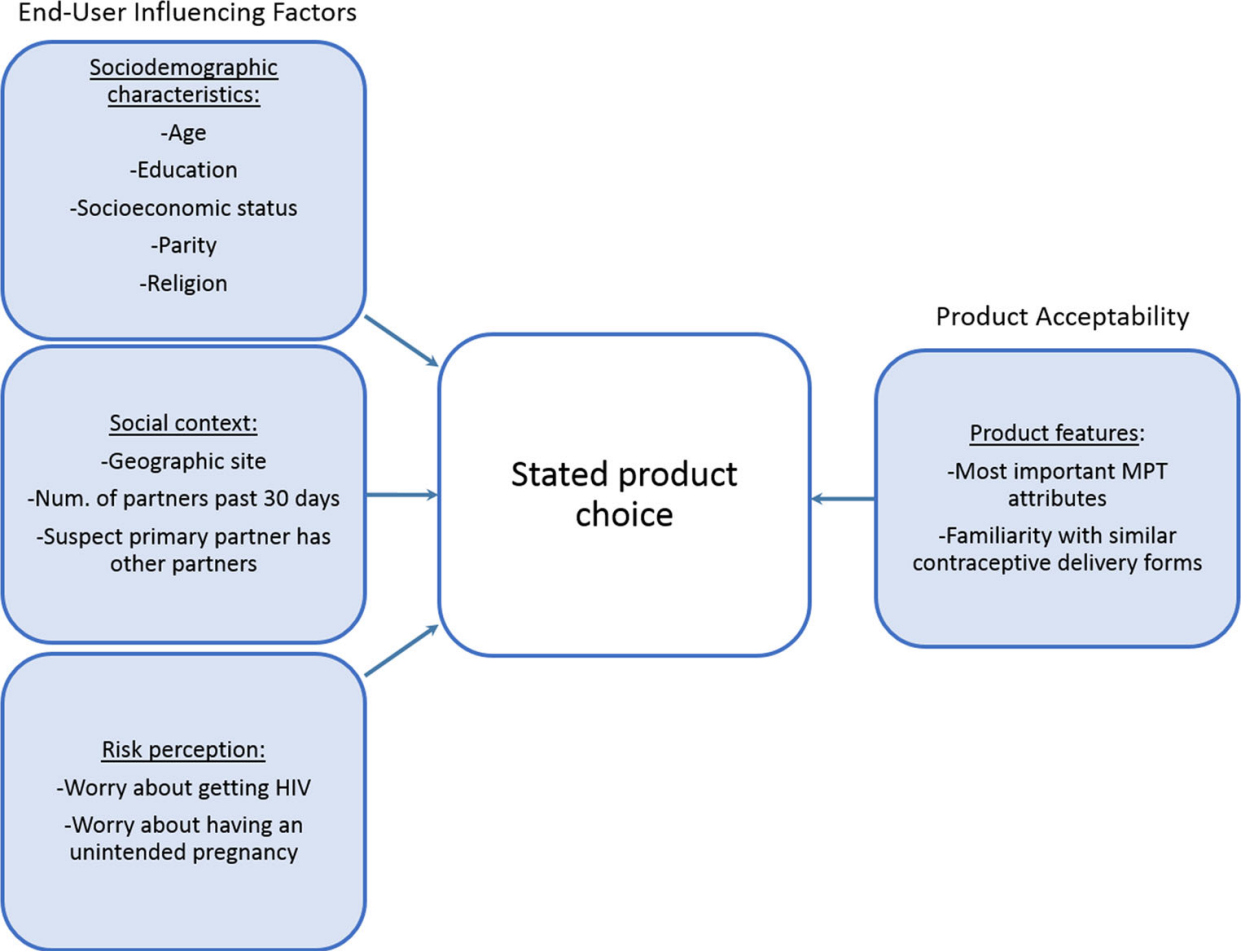
Conceptual framework and domains of hypothesized factors influencing stated product preference

Informed by the conceptual model proposed by Mensch, et al. [11], we considered four domains of potential drivers of stated product choice within the broad areas of end-user influencing factors and product acceptability: (1) sociodemographic characteristics; (2) social context; (3) risk perception; and (4) product features (see Fig. 2).

Sociodemographic characteristics included age, educational attainment, food insecurity (based on participant report of how often in the past 4 weeks she worried she would not have enough food), parity, and religion. Worry about food insecurity was selected as a proxy socio-economic status measure due to evidence of its relationship with HIV risk factors [25, 26], and because of its variation within sites relative to other socio-economic characteristics measured, such as household assets, which tended to vary only between sites. Based on previous studies that have shown differences in preference and behavior between younger and older women, we hypothesized that age would be an important influencing factor for stated product choice. Age was dichotomized into two groups, ages 18–24 and 25–30, to align with the United Nations definitions of youth [27].

We considered women’s perception of risk for both HIV infection and unplanned pregnancy, as measured by asking how worried they were that they might get HIV in the next 12 months, and how likely it was they would have an unplanned pregnancy in the next 12 months, respectively. Response options to these two questions corresponded with five-point Likert scales.

Hypothesized influencers in the social context domain included geographic site (Soshanguve, South Africa, or Kisumu, Kenya) and two measures related to women’s partnerships: the number of sexual partners in the past 30 days; and whether they suspected their primary sexual partner had other partners.

Product features hypothesized to be influential included two factors: MPT attributes named as important, and familiarity with similar (contraceptive) delivery forms. Women were asked to free list the considerations they felt would be most important and second most important when choosing an MPT product, and responses were later coded into categories using codes generated through formative research and edited iteratively throughout the study. For this analysis, we included attributes linked to and varying across the specific delivery forms as explanatory variables: potential side effects/safety, availability/access, and frequency of use. Note that most participants who listed frequency did not specify a preference for high versus low frequency, though one woman mentioned wanting a product you don’t have to take every day, and two women expressed interest in a product that would only need to be used once. Familiarity with similar delivery forms/methods was measured through prior use of long-acting reversible methods (contraceptive implants/IUD) and of injections, prior use of contraceptive pills, and experience with inserting fingers, tampons, or other materials into the vagina for any purpose in the past 3 months (an insertion procedure required for the TRIO ring).

Finally, the two outcomes examined were product approval rating and stated product choice. Product approval rating was assessed for each product by asking, “On a scale of 1–5, how much would you like (taking the tablets/receiving injections/using the ring) for both pregnancy and HIV prevention?” with response options ranging from “1: dislike very much” to “5: like very much”. Stated choice for the tablets, injections, or ring was assessed by asking “If you could choose one of these products now to use for both pregnancy and HIV prevention, which one would it be?” Both approval rating and stated choice were collected before and after showing the educational video.

### Analysis

For all analyses, the five-point Likert-scale risk perception measures were recoded into dichotomous variables based on the distribution of responses. We described characteristics of the study sample by site, and presented product approval ratings graphically to examine the overall distributions of approval scores for each product. Next, we explored the relationship between stated choice (for the ring, tablets or injections) and various end-user and product acceptability factors hypothesized to influence acceptability using the Pearson’s Chi square test. We also examined the shape of the relationship between age and product choice graphically to determine if this relationship differed between younger and older women, to ensure the appropriateness of our age categories.

Finally, we performed multinomial logistic regression analysis to calculate adjusted relative risk ratios (aRRR) of stated product choice associated with the factors hypothesized in our conceptual model to influence stated choice. Multivariable analyses included factors significantly associated with product choice in bivariate analyses at the p < 0.2 level. We explored possible geographic site differences in important factors by running the adjusted models by site. For factors whose relative risk ratios differed in direction and significance between the two sites, we tested for significant interaction with site at p < 0.2. In the final model, factors associated with stated choice at p < 0.05 were considered statistically significant.

## Results

Key baseline characteristics of the 277 women enrolled in TRIO are presented in Table 1. At both sites, participants’ ages were evenly distributed across the age range of 18 and 30, with a median age of 23.5. Approximately two-thirds of the sample was composed of young women aged 18–24.

**Table 1:** Baseline characteristics of women participating in the Trio Study, Soshanguve, South Africa and Kisumu, Kenya, 2015–2016

	Soshanguve (n = 140)	Kisumu (n = 137)	Overall (n = 277)
Age			
Mean, median (min–max)	23.8, 23.7 (18.2–30.6)	23.7, 23.4 (18.2–30.4)	23.8, 23.5 (18.2–30.6)
18–24	92 (65.7)	91 (66.4)	183 (66.1)
25–30	48 (34.3)	46 (33.6)	94 (33.9)
Marital status			
Legally or traditionally married	5 (3.6)	65 (47.5)	70 (25.3)
Not married	135 (96.4)	72 (52.6)	207 (74.7)
Highest level of education			
Attended or completed primary school	1 (0.7)	36 (28.3)	37 (13.4)
Secondary school, not complete	53 (37.9)	44 (32.1)	97 (35.0)
Secondary school, complete	64 (45.7)	49 (35.8)	113 (40.8)
Attended college or university	22 (15.7)	8 (5.8)	30 (10.8)
Religion			
Christian	122 (87.1)	121 (88.3)	243 (87.7)
Muslim	0 (0)	14 (10.2)	14 (5.1)
None	18 (12.9)	2 (1.5)	20 (7.2)
Food insecurity			
Never	86 (61.4)	42 (30.7)	128 (46.2)
Rarely or sometimes	36 (25.7)	72 (52.6)	108 (39.0)
Often	18 (12.9)	23 (16.8)	41 (14.8)
Parity			
Median (min–max)	1.0 (0–4)	1.0 (0–4)	1.0 (0–4)
0	31 (22.1)	30 (21.9)	61 (22.0)
1 or more	109 (77.9)	107 (78.1)	216 (78.0)
Contraceptive methods ever used^a^			
Male condom	131 (93.6)	124 (90.5)	255 (92.1)
Female condom	7 (5.0)	18 (13.1)	25 (9.0)
Pills	33 (23.6)	39 (28.5)	72 (26.0)
Implants	36 (25.7)	62 (45.3)	98 (35.4)
Injectable	113 (80.7)	81 (59.1)	194 (70.0)
IUD	7 (5.0)	7 (5.1)	14 (5.1)
Diaphragm/gel	0 (0.0)	2 (1.5)	2 (0.7)
Traditional/rhythm method	1 (0.7)	5 (3.7)	6 (2.2)
None	1 (0.7)	1 (0.7)	2 (0.7)
Current contraceptive methods^a^			
Male condom	80 (57.1)	61 (44.5)	141 (50.9)
Female condom	2 (1.4)	6 (4.4)	8 (2.9)
Pills	9 (6.4)	9 (6.6)	18 (6.5)
Implants	30 (21.4)	37 (27.0)	67 (24.2)
Injectable	74 (52.9)	41 (29.9)	115 (41.5)
IUD	7 (5.0)	4 (2.9)	11 (4.0)
Diaphragm/gel	0 (0.0)	1 (0.7)	1 (0.4)
Traditional/rhythm method	1 (0.7)	3 (2.2)	4 (1.4)
None	12 (8.6)	17 (12.4)	29 (10.5)
Relationship status			
Currently has primary partner	135 (96.4)	126 (92.0)	261 (94.2)
Does not have primary partner	5 (3.6)	11 (8.0)	16 (5.8)
Number of partners past 30 days			
0	2 (1.4)	10 (7.3)	12 (4.3)
1	127 (90.7)	106 (77.4)	233 (84.1)
More than 1	11 (7.9)	21 (15.3)	32 (11.6)
Exchange sex ever			
Yes	8 (5.7)	25 (18.3)	33 (11.9)
No	132 (94.3)	112 (81.8)	244 (88.1)

^a^Does not sum to 100% as participants could indicate more than one option

Nearly all women (96%) in Soshanguve were not married, compared with approximately half (53%) of women in Kisumu, though the majority of women at both sites indicated currently having a primary partner (96% in Soshanguve and 92% in Kisumu). Of women who reported having a primary partner, the median relationship duration was 3 years at both sites, with one-quarter in relationships of short duration (less than 1 year) and one-quarter in relationships of longer duration (more than 5 years). Women in Kisumu had lower educational attainment than women in Soshanguve. Parity was similar at both sites, with most women (78%) having given birth at least once. Besides condoms, with which nearly all women had experience, the most common forms of contraception ever used among study participants were injectables (81% in Soshanguve and 59% Kisumu), and implants (26% in Soshanguve and 45% in Kisumu).

### Effect of Educational Video

Overall, there was little change in either stated choice or product approval ratings between the pre- and post-video measurements. The only significant change was the approval of the ring, which increased by a mean of 0.35 points (p < 0.001). Therefore, for all subsequent analyses, we selected as outcomes the measures collected after showing the video to increase standardization of basic knowledge on product dosing and use attributes.

### Approval Rating for Each Product: Descriptive Results

**Fig. 3:**
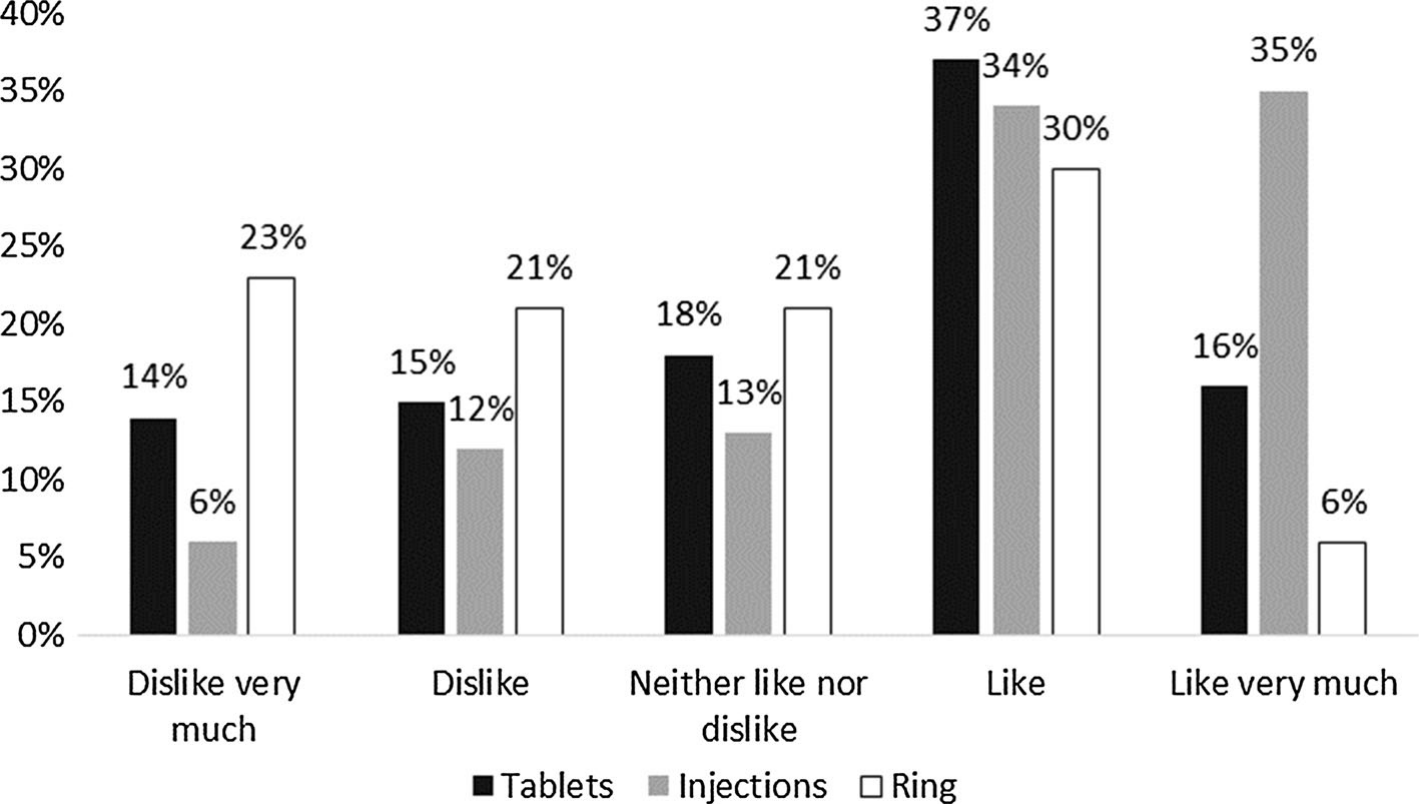
Approval Ratings by Product

Figure 3 presents the distribution of approval ratings on a scale of 1–5 for each product. Nearly one third (29%) of women indicated feeling neutral about or liking/liking very much all three products, though more women (35%) said they liked the injections very much, while women tended to give lower ratings to the ring, with 44% saying they either disliked it or disliked it very much. When rating the three products, the majority (52%) exhibited large variation in their approval ratings across the products, with either 3 or 4 points difference between their lowest and highest rated products. Very few (8%, n = 21) gave all three products the exact same rating. Of these, the majority (n = 18) said they liked all three products (gave a rating of 4 out of 5).

### Stated Choice Among the Three Products: Relationship to Influencing Factors

Of the 277 participants, the majority (62%, 172 women) said they would choose the injections, followed by 75 (27%) who would choose the tablets and 30 (11%) who would choose the ring. Table 2 presents characteristics hypothesized to be important influences on acceptability, by stated product choice. In these bivariate analyses, geographic site, age, worry about getting infected with HIV in the next 12 months, perceived likelihood of having an unintended pregnancy in the next 12 months, the importance of side effects, availability, frequency of use, and past use of contraceptive pills, implants/IUD, injectables, and no family planning ever were all associated with stated product choice (p < 0.2) from the Pearson Chi squared test. Graphs of age and the likelihood of choosing each product suggested linearity between age and product choice, with a slight inflection point at age 25, corresponding to our chosen cut-point. No family planning ever was not included as a variable in the adjusted models due to empty cells.

**Table 2:** Hypothesized influencing factors and stated product choice among women participating in the Trio Study

	Tablets (n = 75; 27.1%)	Injections (n = 172; 62.1%)	Ring (n = 30; 10.8%)	Pearson Χ^2^ *p* value
*Sociodemographic factors*				
Age**				0.031
18–24	58 (31.7)	109 (59.6)	16 (8.7)	
25–30	17 (18.1)	63 (67.0)	14 (14.9)	
Highest level of education				0.334
Attended or completed primary school	10 (27.0)	19 (51.4)	8 (21.6)	
Secondary school, not complete	25 (25.8)	64 (66.0)	8 (8.3)	
Secondary school, complete	33 (29.2)	68 (60.2)	12 (10.6)	
Attended college or university	7 (23.3)	21 (70.0)	2 (6.7)	
Religion				0.270
Christian	64 (26.3)	152 (62.6)	27 (11.1)	
Muslim	5 (35.7)	6 (42.9)	3 (21.4)	
None	6 (30.0)	14 (70.0)	0 (0)	
Worry not enough food in past 4 weeks				0.436
Never	40 (31.3)	78 (60.9)	10 (7.8)	
Rarely or sometimes	26 (24.1)	67 (62.0)	15 (13.9)	
Often	9 (22.0)	27 (65.9)	5 (12.2)	
Parity				0.935
0	16 (26.2)	39 (63.9)	6 (9.8)	
1 or more	59 (27.3)	133 (61.6)	24 (11.1)	
*Contextual factors*				
Geographic site*				0.079
Soshanguve	31 (22.1)	96 (68.6)	13 (9.3)	
Kisumu	44 (32.1)	76 (55.5)	17 (12.4)	
Number of sex partners past 30 days				0.584
0	4 (33.3)	7 (58.3)	1 (8.3)	
1	62 (26.6)	148 (63.5)	23 (9.9)	
More than 1	9 (28.1)	17 (53.1)	6 (18.8)	
Primary partner has other sex partners				0.945
Yes, I know	9 (25.0)	23 (63.9)	4 (11.1)	
Yes, I suspect or Don’t know	41 (28.3)	88 (60.7)	16 (11.0)	
No	21 (26.3)	52 (65.0)	7 (8.8)	
No primary partner	4 (25.0)	9 (56.3)	3 (18.8)	
Exchange sex ever				0.308
Yes	7 (21.2)	20 (60.6)	6 (18.2)	
No	68 (27.9)	152 (62.3)	24 (9.8)	
*Risk perception*				
Worry about HIV infection next 12 months†				0.181
Not at all/a little worried	42 (23.5)	116 (64.8)	21 (11.7)	
Somewhat/very/extremely worried	33 (33.7)	56 (57.1)	9 (9.2)	
Unintended pregnancy next 12 months**				0.046
Extremely/very unlikely	53 (24.4)	143 (65.9)	21 (9.7)	
Somewhat/very/extremely likely	22 (36.7)	29 (48.3)	9 (15.0)	
*Product features*				
Most/second most important attribute				
Side effects/safety†				0.155
Yes	26 (23.2)	77 (68.8)	9 (8.0)	
No	49 (29.7)	95 (57.6)	21 (12.7)	
Availability/access*				0.053
Yes	23 (39.7)	30 (51.7)	5 (8.6)	
No	52 (23.7)	142 (64.8)	25 (11.4)	
Frequency of use**				<0.001
Yes	7 (10.9)	53 (82.8)	4 (6.3)	
No	68 (31.9)	119 (55.9)	26 (12.2)	
Experience with vaginal insertion				0.235
Yes	26 (21.9)	79 (66.4)	14 (11.8)	
No	49 (31.0)	93 (58.9)	16 (10.1)	
Contraceptive methods ever used				
Male condom				0.744
Yes	68 (26.7)	160 (62.8)	27 (10.6)	
No	7 (31.8)	12 (54.6)	3 (13.6)	
Female condom				0.672
Yes	6 (24.0)	15 (60.0)	4 (16.0)	
No	69 (27.4)	157 (62.3)	26 (10.3)	
Pills†				0.133
Yes	26 (36.1)	39 (54.2)	7 (9.7)	
No	49 (23.9)	133 (64.9)	23 (11.2)	
Implants/IUD**				0.012
Yes	25 (32.1)	40 (51.3)	13 (16.7)	
No	50 (25.1)	132 (66.3)	17 (8.5)	
Injectable**				0.003
Yes	44 (22.7)	133 (68.6)	17 (8.8)	
No	31 (37.4)	39 (47.0)	13 (15.7)	
None*				0.066
Yes	2 (100.0)	0 (0)	0 (0)	
No	73 (26.7)	172 (62.6)	30 (10.9)	
Current contraceptive methods				
Male condom				0.922
Yes	39 (27.7)	86 (61.0)	16 (11.4)	
No	36 (26.5)	86 (63.2)	14 (10.3)	
Female condom				0.569
Yes	2 (25.0)	6 (75.0)	0 (0.0)	
No	73 (27.1)	166 (61.7)	30 (11.2)	
Pills*				0.083
Yes	7 (38.9)	7 (38.9)	4 (22.2)	
No	68 (26.3)	165 (63.7)	26 (10.0)	
Implants/IUD**				0.040
Yes	25 (32.1)	40 (51.3)	13 (16.7)	
No	50 (25.1)	132 (66.3)	17 (8.5)	
Injectable**				<0.001
Yes	18 (15.7)	88 (76.5)	9 (7.8)	
No	57 (35.2)	84 (51.9)	21 (13.0)	
None				0.391
Yes	9 (31.0)	19 (65.5)	1 (3.5)	
No	66 (26.6)	153 (61.7)	29 (11.7)	

Row percentages presented

†p < 0.20; *p < 0.10; **p < 0.05

Table 3 presents results of a multinomial logistic regression analysis examining factors associated with product preference. In analyses stratified by site, there were differences in the relationship between age and product preference, though the interaction was not statistically significant, so combined results are presented in the table and those differences are described in text. HIV and pregnancy risk perception were not significantly associated with stated product choice.

**Table 3:** Adjusted relative risk ratios for stated product choice among women participating in the Trio Study

N = 277	Tablets (vs injections) aRRR (95% CI)	Ring (vs injections) aRRR (95% CI)	Ring (vs tablets) aRRR (95% CI)
Site			
Soshanguve	[ref]	[ref]	[ref]
Kisumu	0.90 (0.40–2.03)	0.49 (0.15–1.63)	0.55 (0.15–1.98)
Age			
17–24	[ref]	[ref]	[ref]
25–30	0.57 (0.29–1.12)	1.74 (0.73–4.15)	3.07 (1.15–8.17)**
Worry about HIV infection			
Not at all/a little worried	[ref]	[ref]	[ref]
Smwht/very/extremely worried	1.60 (0.87–2.96)	0.81 (0.33–2.00)	0.50 (0.19–1.32)
Unintended pregnancy next 12 months			
Extremely/very unlikely	[ref]	[ref]	[ref]
Somewhat/very/extremely likely	1.46 (0.72–2.96)	2.08 (0.79–5.47)	1.43 (0.51–4.00)
Most/second most important attributes			
Side effects	0.89 (0.40–1.97)	0.40 (0.12–1.32)	0.45 (0.12–1.64)
Availability	1.41 (0.66–3.00)	0.48 (0.15–1.51)	0.34 (0.10–1.12)
Frequency	0.27 (0.11–0.67)***	0.26 (0.08–0.92)**	0.99 (0.23–4.22)
Prior contraceptive use			
Pills	1.92 (1.01–3.67)**	0.94 (0.35–2.53)	0.49 (0.17–1.39)
Injectables	0.52 (0.27–1.01)	0.38 (0.16–0.94)**	0.74 (0.28–1.93)
Implants/IUD	1.08 (0.58–2.02)	3.13 (1.31–7.48)**	2.88 (1.12–7.45)**

*** p < 0.01, ** p < 0.05

Age was an important predictor of choosing the ring over the tablet, though this result was driven by age differences at the Kenya site. Overall, women aged 25–30 were three times more likely to prefer the ring over the tablet, compared to women aged 18-24 (aRRR = 3.1; 95% CI 1.2–8.2). In site-specific models, age was not significantly associated with product preference in Soshanguve, while in Kisumu, older women were significantly more likely to prefer the ring over the tablets (aRRR = 5.7; 95% CI 1.4–23.4).

Prior experience with similar contraceptive delivery forms was related to which MPT product women said they would choose. Women who had experience with contraceptive pills were more likely to prefer the tablets over the injection (aRRR = 1.9; 95% CI 1.0–3.7), and women who had experience with injectable contraceptives were less likely to prefer rings compared with injections (aRRR = 0.4; 95% CI 0.2–0.9). Women who had used long-acting reversible contraceptive methods (LARCs) (contraceptive implants or an IUD) were significantly more likely to prefer the ring over the other two products (aRRR = 3.1; 95% CI 1.3–7.5) compared to injections and compared to tablets (aRRR = 2.9, 95% CI 1.1–7.5).

The product features that women listed as important or most important aligned with the key attributes of the product they stated they would choose. Women for whom frequency of use was an important attribute were less likely to prefer the tablets (aRRR = 0.3; 95% CI 0.1–0.7), which has the highest dosing frequency, or the ring (aRRR = 0.3; 95% CI 0.1–0.9), compared to the injections.

## Discussion

In the baseline visit of the TRIO Study, product-naïve women in Kisumu, Kenya and Soshanguve, South Africa were asked to state their choice among three hypothetical MPT delivery forms after viewing a brief standardized educational video on the three products: oral tablets, vaginal ring, and injections. They also provided their approval rating of each delivery form. The majority of women in the study indicated they would choose to use an injectable product to prevent HIV and pregnancy over oral tablets or a vaginal ring. However, the results highlight several key differences between women who preferred the injections and those—in the minority—who preferred the daily tablets or a monthly vaginal ring.

### Stated Choice of Injections

Women who had used injectable contraception were more likely to indicate a stated choice for the injections relative to either the ring or the tablets, likely because their familiarity with injections influenced a preference for an injectable MPT. Women who indicated frequency of use as an important product attribute were less likely to prefer the tablets and the ring relative to the injections, which provide an advantage in terms of low frequency of use, low user burden and correct administration (by a provider) [28]. However, it is worth noting that a much higher proportion of participants reported having ever used injectable contraception than were currently using the method (70% vs. 42%), indicating they had discontinued or switched away from this method of family planning. Many women switch or discontinue use of contraceptive methods due to side effects, contextual factors, and other reasons [29, 30] that our study was not able to incorporate given the hypothetical nature of the MPT product and our focus on acceptability of the delivery form, and not the drug(s) they may eventually contain.

### Stated Choice of Tablets

About a quarter of the women in the TRIO Study indicated they would choose the oral tablets for HIV and pregnancy prevention. The main predictor of choosing tablets over injections was prior use of pills for contraception, as these women have experienced having to take a pill every day and may also have been successful in the past at daily pill taking. Though most of the TRIO participants had likely experienced taking oral medication tablets at some point in their lives, the daily dosing requirement as a prevention behavior sets oral PrEP apart from daily oral dosing to treat a disease. In addition, the Truvada pill’s large size and its resemblance to an ARV and the associated potential for stigma, concerns that have been expressed by women in other studies [31, 32], may have decreased interest in this delivery form.

### Stated Choice of the Ring

Overall, of the three delivery forms evaluated in the TRIO Study, the vaginal ring was the most novel, the contraceptive Nuvaring having been recently introduced with very low coverage in South Africa [33] and used only in a demonstration study in Kisumu [34]. It was also the least popular product among young women at the baseline visit. Notably, the ring is the most advanced co-formulated MPT in the product development pipeline, so particular emphasis should be given to ways to improve its future acceptability.

Women age 25 and above were three times more likely to prefer the ring than women under 25. This finding aligns with results from the ASPIRE vaginal ring trial, where younger women exhibited lower adherence to the ring relative to older women [18]. The association was driven by data from the Kisumu site, though we suspect the site difference may be due to very few women overall preferring the ring in Soshanguve. Older women may have more stable relationships and therefore may be more willing to disclose the ring to their partners, which may be perceived as the most difficult of the three product to use secretly. Regarding disclosure, women in a mixed methods study of vaginal ring acceptability in South Africa have expressed that married women should disclose product use, but that casual partners could use a product in secret [35]. Of note, more women in Kisumu were married than in Soshanguve, so marital status may have played a role in the site difference.

Women who had used implants or an IUD for contraception were more likely to prefer the ring than their counterparts who had never used those LARCs, which currently have limited provision in SSA [36], although implant use was high in our study population. Women who adopted these methods may represent early adopters of new health technology, and may be more willing to use other novel technologies like the ring. Women who have selected implants or IUDs as a family planning method may also be more comfortable with using a device that remains inside the body for an extended time period. These results highlight the importance of familiarity in selecting a novel product, echoing previous findings from studies that have introduced vaginal rings. Indeed, in qualitative work conducted as part of the ASPIRE ring trial, the majority of women participating in in-depth-interviews at the exit visit, with 12–33 months of experience using the ring, preferred the ring among eight HIV prevention products discussed. Familiarity was cited by these participants as a key reason for this preference [37]. A study in Australian women predicted that the vaginal contraceptive ring, a novel product that had not yet been introduced in that country, would only capture 3% of the contraceptive market share [38] based on a survey that described the product. However, in trials of the contraceptive ring in Europe and North America, familiarity was an important predictor of method preference, with 81% of women perceiving the ring as the best contraceptive method after 3 months of use, in contrast to 66% perceiving the tablet as the best method at baseline [39].

### Limitations and Future Research Directions

Investigating factors related to preference for the less popular products, particularly the ring, was limited by a small sample size of women who selected this as their preferred product at baseline. In addition, a quantitative study may not fully capture nuanced reasons for product choice, particularly in terms of likes and dislikes of various product attributes. At baseline, we collected what women stated they would choose, but did not assess the reason(s) for their stated choice. Comprehensive data examining MPT acceptability and product preference, including several rounds of qualitative interviews and focus groups in the TRIO Study during follow-up will permit in-depth analysis of women’s preferences and use experiences.

A major limitation of the TRIO Study’s ability to draw conclusions about potential uptake of real products is the hypothetical nature of the MPT products. The goal of TRIO was to gather information about acceptability of each delivery form, separate from the active medications that they may eventually contain, but this separation leaves out details that could be important influencers of product preference and uptake. Importantly, the two-injection regimen used in TRIO is significantly simpler than that of the current long-acting injection in phase II that requires lead-in and lead-out dosing of oral PrEP [19], and we did not assess the likely significant impact of this requirement on potential acceptability. In addition, participants were given no information about the possible side effects of each product. Nevertheless, we feel our findings are still informative as the study mimics a real-world scenario in which potential end users are briefly informed about three options and must make a choice, without necessarily having complete information or understanding. Subsequent analysis of prospective data from TRIO will allow us to evaluate whether these preferences shift after opportunities to actually use the products, and to explore the factors influencing any changes observed.

Finally, the role of health care providers and other key informants in product roll-out and uptake cannot be ignored [40, 41], so perspectives of these key informants should also be incorporated into overall decisions about product development and messaging.

### Implications for MPT Research and Development

These results provide a strong case for capitalizing on the acceptability and high uptake of existing contraceptive delivery forms in developing acceptable MPT products. Research will need to identify ways to increase familiarity of women with novel delivery forms, such as the ring, to improve uptake and consistent use in effectiveness trials. Enhanced ring messaging will be needed to engage end-users, particularly younger women in trying this unfamiliar method, which was the least popular of the three delivery forms in this study. Strategies in oral MPT tablet research should incorporate simple strategies to decrease the burden of daily dosing.
